# Prescription profile of Chinese herbal products containing coumestrol, genestein, and/or daidzein among female users: an analysis of national health insurance data in Taiwan between 1997 and 2007

**DOI:** 10.1186/1749-8546-7-22

**Published:** 2012-10-16

**Authors:** Chien-Tung Wu, Jeng-Nan Tzeng, Jung-Nien Lai, Shun-Hua Tsan, Jung-Der Wang

**Affiliations:** 1 Department of Chinese Medicine, Linsen (Chinese Medicine) Branch and Chinese Medicine Clinic Center, Taipei City Hospital, Taipei, Taiwan; 2 Institute of Traditional Medicine, School of Medicine, National Yang-Ming University, Taipei, Taiwan; 3 Department of Mathematical Sciences, National Chengchi University, Taipei, Taiwan; 4 Department of Obstetrics and Gynecology and Department of Chinese Medicine, Yangming Branch, Taipei City Hospital, Taipei, Taiwan; 5 Department of Obstetrics and Gynecology, Zhongxing Branch, Taipei City Hospital, Taipei, Taiwan; 6 Department of Public Health, College of Medicine, National Cheng Kung University, Tainan, Taiwan; 7 Departments of Occupational and Environmental Medicine and Internal Medicine, National Cheng Kung University Hospital, Tainan, Taiwan

## Abstract

**Background:**

Some Chinese herbs contain several kinds of phytoestrogens, and these herbs are commonly prescribed in Taiwan. Phytoestrogens may influence the effects of estrogen in females, although their activities are weak. This study aims to identify the risk and analyze the prescription profile of commonly used phytoestrogenic herbs in Taiwan.

**Methods:**

The study analyzed women who had been prescribed phytoestrogenic herbs including coumestrol, genistein and/or daidzein between 1997 and 2007 in a fixed cohort taken from all female beneficiaries from the National Health Insurance Research Database of Taiwan. The prescription frequencies, cumulated dosages, and primary indications were listed.

**Results:**

A total of 462,861 women were included in the study, of whom ~47.0% had used phytoestrogenic herbs at least once during the study period. A total of 6,270,813 prescriptions were recorded, and more than 20% of these contained phytoestrogens. The most commonly prescribed herb and formula were *Puerariae Radix* and *Ge gen tang (Pueraria Decoction)*, respectively. Most of the prescriptions were issued for diseases of the respiratory system, followed by symptoms, signs, and ill-defined conditions and diseases of the musculoskeletal system and connective tissue.

**Conclusion:**

This study shows that women who sought medical treatment from Chinese medicine doctors for relief of respiratory discomfort had a high possibility of exposure to phytoestrogenic herbs. Safety issues related to the female endocrine system should be a priority for future research.

## Background

Phytoestrogens may interact with the estrogen receptors that mediate endocrine homeostasis, causing damage to reproductive health, especially at developing life stages [1-3]. Although the phytoestrogenic activities are weaker than those of human endogenous estrogens, the consumption of phytoestrogens may have clinically significant consequences [4]. Coumestrol, genistein and daidzein are strong phytoestrogens that are found in many foods and Chinese medicine (CM) product [5-7]. There is a concern among CM doctors and CM users about inconclusive safety results from various *in vitro* and *in vivo* models in estrogenic studies, especially for patients taking Chinese herbal products (CHPs) containing coumestrol, genistein, and/or daidzein (CGD-CHPs).

CM has been an important part of healthcare in Taiwan for hundreds of years, and CHPs have been regularly reimbursed by the National Health Insurance (NHI) system since 1995 [8]. All CHPs are fully reimbursed under the current NHI system of Taiwan, and the claim database provides a platform for understanding the utilization of CGD-CHPs.

This study aims to analyze random samples from the NHI database to determine the risk and prescription profile of CGD-CHPs among female beneficiaries in Taiwan between January 1997 and December 2007.

## Methods

### Study subjects

The source population for this study was women who had been prescribed CGD-CHPs between 1997 and 2007 in a randomly sampled cohort from all NHI female beneficiaries in Taiwan. This research included females from the randomly sampled cohort aged 1 to 99 years as the study subjects, and age was calculated by subtracting the subject’s birthday from the 1^st^ of July of each year. The NHI system provides universal health insurance coverage, and covered more than 97% of the Taiwanese population in 2002 [9]. Consequently, data from the NHI system are widely used by researchers in various fields [10]. Both CM and Western medicine doctors must follow a standard diagnostic procedure using the International Classification of Diseases, 9th Revision, Clinical Modification (ICD-9-CM) coding system for claiming reimbursement. Because the NHI system of Taiwan does not reimburse the use of CM for inpatient services, we only investigated the use of CM for outpatient services. The NHI reimbursement data files were transformed into a research database entitled the National Health Insurance Research Database (NHIRD) that was maintained by the National Health Research Institutes. In the NHIRD, all the identification information is encrypted but the medical records files are retained, including information from 1997 to 2007 on medical care facilities and specialties, drugs and/or treatment regimens (dosages, dosage frequency and prescription duration), patient sex and date of birth, date of visit, and three major diagnoses coded in the ICD-9-CM format. The NHIRD contains a one-million cohort systematically and randomly selected from the 23 million people in the NHI database who were ever insured under the NHI system in 2005. For each subject, we collected all records of CHPs during 1997 to 2007 as a female cohort. We considered the first diagnosis to be the major diagnosis on the records for outpatient department visits, which were coded in ICD-9-CM and then lumped together into different broader disease categories, *e.g.*, lists of ICD-9-CM codes 710–739 were classified as diseases of the musculoskeletal system and connective tissue, while ICD-9-CM codes 460–519 were classified as diseases of the respiratory system.

### List of licensed CHPs

The list of reimbursed CHPs was obtained from the Bureau of the NHI website. The corresponding drug information about specific mixtures or names was obtained from the Committee on Chinese Medicine and Pharmacy (CCMP) website, and included the proportions of each constituent, date, approval period as a drug, code and manufacturers’ names. The CCMP list shows that 10,413 CHPs were licensed during the study period, of which 10,309 CHPs were covered by the NHI system. All CHPs with the same CCMP standard formula were classified under the same category, regardless of slight variations among products of different pharmaceutical companies [11]. For example, there are 62 approved licenses for the formula *Ge gen tang* containing coumestrol, genistein and/or daidzein.

### Selection of herbs and estimation of cumulative doses

The CGD-CHPs examined in this study included *Psoraleae Fructus*, *Puerariae Radix*, *Sojae Semen Praeparatum*, *Sophorae Flavescentis Radix* and *Sophorae Flos*. We examined all of the CHPs licensed by the CCMP between 1997 and 2007, including single herbs and herbal formulae, to determine whether they included CGD-CHPs. The original weight of each herb was then calculated according to the contents manufactured by the individual pharmaceutical company registered for each licence and approved by the CCMP.

### Statistical analysis

The data were analyzed by descriptive statistics, including the decomposition of the coumestrol, genistein and daidzein contents of the licensed and prescribed CHPs, CGD-CHP prescription rates stratified by age, the medians (with 10th and 90th percentiles) of the cumulative doses of coumestrol, genistein and daidzein, population distribution of patients who had been potentially exposed to coumestrol, genistein and daidzein under various dosages, the frequencies of the disease categories prescribed with CGD-CHPs, the most frequently prescribed herbal formulae containing CGD-CHPs, and so on. All of these analyses were performed using the SAS software package (version 9.1; SAS Inc., Cary, NC, USA).

## Results

Between 1 January 1997 and 31 December 2007, 721 (7%) CGD-CHPs were identified among the 10,309 licensed CHPs, of which the top three CGD-CHPs were *Puerariae Radix* (4.9%), *Sophorae Flavescentis Radix* (0.7%), and *Sojae Semen Praeparatum* (0.7%). A total of 6,270,813 cases of prescribed and reimbursed CGD-CHPs were recorded (Table 1). Among all the CGD-CHPs, *Puerariae Radix* was the most frequently prescribed as a single herb and in the formulae by CM practitioners in Taiwan. The co-existence of more than two major active phytoestrogens was identified in licensed and prescribed CGD-CHPs. Genistein-containing herbal prescriptions comprised 80% of the prescribed CGD-CHPs.

**Table 1:** Distribution frequencies of licensed and prescribed CHPs containing phytoestrogens* based on a one-million patient random sample (1997–2007) from the NHI system of Taiwan

**Chinese herbal products**	**No. (%) of licensed CHPs**	**No. (%) of female CHP users**	**No. (%) prescriptions of CHPs**	**Cumulative dose (g) median (10%-90%)**	**Major corresponding (metabolic) active substance(s) in humans**
Total no. (%) licensed CHPs	10,309 (100)	342,440 (100)	6,270,813 (100)	-	
Licensed CGD-CHPs	721 (7.0)	217,897 (63.6)	1,389,244 (22.2)	-	
*Puerariae Fructus*	60 (0.6)	13,761 (4.0)	35245 (0.6)	5.9 (2.0-34.0)	Genistein, daidzein and biochanin A, coumestrol
*Puerariae Radix*	505 (4.9)	182,899 (53.4)	986,110 (15.7)	10.8 (1.7-57.6)	Puerarin, daidzin, genistin, daidzein and genistein, coumestrol
*Sojae Semen Praeparatum*	74 (0.7)	86,273 (25.2)	323,413 (5.2)	4.2 (0.6-17.5)	Genistein, genistin, daidzein, daidzin, glycitein and glycitin, coumestrol
*Sophorae Flavescentis Radix*	75 (0.7)	63,511 (18.5)	180,720 (2.9)	4.9 (1.5-15.0)	Daidzein, genistein and formononetin
*Sophorae Flos*	38 (0.4)	8,853 (2.6)	25,871 (0.4)	12.6 (2.0-48.0)	Genistin, genistein

*Phytoestrogens mainly contained coumestrol, genistein and/or daidzein.

Among the one-million randomly sampled cohorts, 462,861 women between the ages of 1 and 99 years were included in the analysis. From 1997 to 2007, 342,440 (74.0%) of the women used CHPs on at least one occasion, of whom 217,897 (63.6%) were treated with prescriptions containing coumestrol, genistein and/or daidzein. The CGD-CHPs-exposed population included many women of reproductive age (Table 2). When calculating individuals’ exposure, most patients were exposed to low cumulative doses (less than 50 g) of CGD-CHPs, although ~5% of the patients were prescribed with *Puerariae Radix* at cumulative doses above 57.6 g (Table 1). Given that the random sample in this cohort accounted for approximately 5% of the Taiwanese population, it can be inferred that about 180,000 women were exposed to such high cumulative doses of *Puerariae Radix*.

**Table 2:** **Prescription frequencies of CHPs containing phytoestrogen**^a^* and cumulative average original weights of the five major Chinese herbs stratified by age, based on a one-million patient random sample (1997–2007) from the NHI system of Taiwan

	**Age**								
**Chinese herbal products**	≤ 9	10-19	20-29	30-39	40-49	50-59	60-69	70-79	≥ 80
Total licensed CHPs	252,268	594,059	1,069,122	1,368,722	1,332,924	818,922	496,445	268,747	69,604
Licensed CGD-CHPs.	62,140	135,716	220,967	307,731	301,586	186,354	108,348	54,060	12,342
**Average dosage per woman-year (g) **^a^									
*Puerariae Fructus*	10.2	18.0	14.5	15.9	17.9	21.3	21.0	22.0	21.3
*Puerariae Radix*	25.8	27.8	29.2	33.7	37.0	39.0	41.4	41.5	40.4
*Sojae Semen Praeparatum*	8.6	9.5	9.6	11.0	11.4	11.4	12.4	11.3	10.5
*Sophorae Flavescentis Radix*	9.0	10.5	10.9	10.9	10.7	10.5	10.8	10.6	11.4
*Sophorae Flos*	15.9	24.6	27.3	33.5	35.5	37.3	36.4	49.9	34.3

^a^ Converted back to the original weight.

*Phytoestrogens mainly contained coumestrol, genistein and/or daidzein.

The major disease categories with CGD-CHPs prescription included respiratory diseases (556,464 visits) and symptoms, signs, and ill-defined conditions (263,742 visits), followed by musculoskeletal system and connective tissue diseases (152,471 visits) and digestive diseases (105,448 visits) (Table 3). CGD-CHPs were usually prescribed in the form of herbal formulae, especially *Ge gen tang (Pueraria Decoction,* containing *Puerariae Radix*) and *Yin qiao san (Lonicera and Forsythia Powder,* containing *Sojae Semen Praeparatum*) (Table 4). Most CGD-CHPs were prescribed for a treatment period of less than 30 days, and the most common dosage frequency was three times per day. Furthermore, most CM female users consumed no more than 30 g of CDG-CHPs containing daidzein and genistein.

**Table 3:** Frequency distribution of the top 10 disease categories for prescribing CHPs containing phytoestrogens*

**Major disease category**	**ICD-9-CM code range**	**Number of visits**	**%**
Total	-	1,389,244	100
Diseases of the respiratory system	460–519	556,464	40.1
Symptoms, signs, and ill-defined conditions	780–799	263,742	19.0
Diseases of the musculoskeletal system and connective tissue	710–739	152,471	11.0
Diseases of the digestive system	520–579	105,448	7.6
Diseases of the skin and subcutaneous tissue	680–709	95,315	6.9
Diseases of the genitourinary system	580–629	65,837	4.7
Injury and poisoning	800–999	39,366	2.8
Diseases of the nervous system and sense organs	320–389	35,585	2.6
Diseases of the circulatory system	390–459	22,932	1.7
Endocrine, nutritional and metabolic diseases, and immunity disorders	240-279	14,889	1.1

The population was women aged 0–99 years among the one-million patient NHIRD cohort.

*Phytoestrogens mainly contained coumestrol, genistein and/or daidzein.

**Table 4:** **Commonly prescribed single herbs and herbal formulae of CGD-CHPs **^**a**^

**Drug name**		**Herbs with phytoestrogens**	**Prescriptions**	**%**
Total herbal prescription			6,270,813	100
Herbal prescription containing coumestrol, genistein and/or daidzein			1,389,244	22.2
***Single herb (Chinese name)***				
*Ge Gen*		*Puerariae Radix*	223,313	3.6
*Bu Gu Zhi*		*Puerariae Fructus*	24,040	0.4
*Ku Shen Gen*		*Sophorae Flavescentis Radix*	17,865	0.3
*Huai Hua*		*Sophorae Flos*	11,811	0.2
*Dan Dou Chi*		*Sojae Semen Praeparatum*	5,132	0.1
***Herbal formulae***	***(English name)***			
*Ge gen tang*	*Pueraria Decoction*	*Puerariae Radix*	315,781	5.0
*Yin qiao san*	*Lonicera and Forsythia Powder*	*Sojae Semen Praeparatum*	307,692	4.9
*Chai ge jie ji tang*	*Bupleurum and Pueraria Flesh-Resolving Decoction*	*Puerariae Radix*	115,629	1.8
*Xiao feng san*	*Wind-Dispersing Powder*	*Sophorae Flavescentis Radix*	85,502	1.4
*Dang gui nian tong tang*	*Chinese Angelica Pain-Assuaging Decoction*	*Puerariae Radix Sophorae Flavescentis Radix*	81,063	1.3
*Shi shen tang*	*Ten Spirits Decoction*	*Puerariae Radix*	75,271	1.2
*Ge gen qin lian tang*	*Pueraria, Scutellaria, and Coptis Decoction*	*Puerariae Radix*	60,686	1.0
*Shen su yin*	*Ginseng and Perilla Beverage*	*Puerariae Radix*	34,848	0.6
*Qing shu yi qi tang*	*Summerheat-Clearing Qi-Boosting Decoction*	*Puerariae Radix*	32,338	0.5
*San zhong kui jian tang*	*Swelling-Dispersing Hardness-Breaking Decoction*	*Puerariae Radix*	27,071	0.4

^a^ CGD-CHPs refers to Chinese herbal products containing coumestrol, genistein and/or daidzein.

## Discussion

To the best of our knowledge, this is the first study to document the use of CHPs containing phytoestrogens in a random national-level sample containing all detailed medical records. We focused on active ingredients that could be considered to be potent phytoestrogens, namely coumestrol, genistein and daidzein, in women who sought medical treatment from CM doctors. Among the licensed CHPs in Taiwan, 7% contained coumestrol, genistein and/or daidzein. However, these products comprised 22.2% of the CM prescriptions consumed by about half of the female population in Taiwan during the 11-year study period. Diseases of the respiratory system were the most frequent disease category for which CGD-CHPs were prescribed, constituting 40% of CM visits. This finding indicates that female CM users in Taiwan may not have expected to be exposed to phytoestrogenic herbs when they used CM therapies to manage the discomfort of a sore throat, cough, and runny nose. Exposure to *Puerariae Radix* was the most extensive, and nearly 5% of female CM users consumed cumulative doses of *Puerariae Radix* above 60 g.

In addition, the present data show that the major consumers of CDG-CHPs were aged between 20 and 49 years. Little is known about phytoestrogenic herbs and their potential interactions with the endocrine system, and therefore special attention should be drawn to the herbal formulae containing phytoestrogenic herbs, especially the prescriptions for female patients of reproductive age suffering from respiratory diseases.

According to CM theory [12], *Puerariae Radix* can dispel exterior cold syndrome of the excessive type and alleviate muscle aches in the neck and back caused by cold constriction. Previous *in vitro* studies have indicated that *Puerariae Radix* is rich in coumestrol, genistein and daidzein as soya, which may reduce cancer cell viability and induce apoptosis [13,14]. And, epidemiological studies also suggested that exposure to relatively high concentrations of phytoestrogens early in life may decrease the prevalence of cancer development later in life [15,16]. CM doctors should observe the potential effects of phytoestrogenic herbs on consumers’ endocrine systems proactively at the early stage to avoid any consequences of chronic CM therapy administration.

Our study has two limitations. First, the NHI system only reimburses CHPs, and decoctions are not included and cannot be generalized for their usage. In addition, this study did not include Chinese herbal remedies and health foods containing herbs that can be directly purchased from CM pharmacies. Thus, the frequency of phytoestrogenic herb consumption might be underestimated. Because the NHIRD collects all prescription information prospectively, we can rule out the possibility of a recall bias concerning intake dosages and different types of herbal prescriptions. In addition, as the rate of insured individuals in the NHIRD has consistently exceeded 96% since 1997, we can exclude the possibility of a selection bias. Therefore, the estimated prevalence rate presented here is close to the true use of CGD-CHPs by female beneficiaries in Taiwan. Another limitation is that only herbs containing coumestrol, genistein and/or daidzein were included in the study, meaning that there must be caution in generalizing these findings to use of phytoestrogens among the female population.

## Conclusion

This study shows that women who sought medical treatment from CM doctors for relief of respiratory discomfort had a high possibility of exposure to phytoestrogenic herbs. Safety issues related to the female endocrine system should be a priority for future research.

## Abbreviations

CM: Chinese medicine; CHP: Chinese herbal products; CGD-CHPs: Chinese herbal products containing coumestrol, genistein and/or daidzein; NHI: National Health Insurance; NHIRD: national health insurance research database; CCMP: Committee on Chinese Medicine and Pharmacy; ICD-9-CM: International Classification of Diseases, 9th Revision, Clinical Modification.

## Competing interests

The sponsors of the study had no role in the study design, data collection, data analysis, data interpretation, or writing of the report. All authors had financial support from the Committee on Chinese Medicine and Pharmacy of the Department of Health (Taiwan), and National Health Research Institutes (Taiwan) for the submitted work; no financial relationships with any organizations that might have an interest in the submitted work in the previous 5 years; and no other relationships or activities that could appear to have influenced the submitted work.

## Authors’ contributions

CTW, JNL, and JDW designed the study. CTW and JNL acquired the data. CTW, JNL, and JDW analyzed the data. CTW and JNL wrote the manuscript. JNT, HST, and JDW revised the manuscript. JDW provided technical advice. All authors read and approved the final version of the manuscript.

## References

[B1] Humfrey CD Phytoestrogens and human health effects: weighing up the current evidence Nat Toxins 1998 6 51 59 10.1002/(SICI)1522-7189(199804)6:2<51::AID-NT11>3.0.CO;2-9 9888630

[B2] Cassidy A Potential risks and benefits of phytoestrogen-rich diets Int J Vitam Nutr Res 2003 73 120 126 10.1024/0300-9831.73.2.120 12747219

[B3] Limer JL Parkes AT Speirs V Differential response to phytoestrogens in endocrine sensitive and resistant breast cancer cells in vitro Int J Cancer 2006 119 515 521 10.1002/ijc.21863 16506217

[B4] Golden RJ Noller KL Titus-Ernstoff L Kaufman RH Mittendorf R Stillman R Reese EA Environmental endocrine modulators and human health: an assessment of the biological evidence Crit Rev Toxicol 1998 28 109 227 10.1080/10408449891344191 9557209

[B5] Takeuchi S Takahashi T Sawada Y Iida M Matsuda T Kojima H Comparative study on the nuclear hormone receptor activity of various phytochemicals and their metabolites by reporter gene assays using Chinese hamster ovary cells Biol Pharm Bull 2009 32 195 202 10.1248/bpb.32.195 19182375

[B6] Harris DM Besselink E Henning SM Go VL Heber D Phytoestrogens induce differential estrogen receptor alpha- or Beta-mediated responses in transfected breast cancer cells Exp Biol Med (Maywood) 2005 230 558 568 16118406 10.1177/153537020523000807

[B7] Lee SH Jung BH Kim SY Chung BC Determination of phytoestrogens in traditional medicinal herbs using gas chromatography–mass spectrometry J Nutr Biochem 2004 15 452 460 10.1016/j.jnutbio.2004.01.007 15302079

[B8] Yang YH Chen PC Wang JD Lee CH Lai JN Prescription pattern of traditional chinese medicine for climacteric women in taiwan Climacteric 2009 12 541 547 10.3109/13697130903060081 19905906

[B9] Chou SY Liu JT Hammitt JK National health insurance and precautionary saving: evidence from taiwan J Health Econ 2003 87 1873 1894

[B10] International publications regarding the use of national health insurance database taiwanhttp://www.nhri.org.tw/nhird/talk_07.htm.

[B11] The national health insurance statistics – beneficiaries profile http://www.nhi.gov.tw/english/webdata.asp?menu=11&menu_id=290&Cmenu_ID=302&webdata_id=2974.

[B12] Cai YM He Y Qiu T Zou J Sun DP Peng QH Jia RX Zhao HR Research on frequency of application with modern chinese herbal medicine Chin J Integr Med 2011 17 64 70 10.1007/s11655-011-0609-2 21258899

[B13] Lin YJ Hou YC Lin CH Hsu YA Sheu JJ Lai CH Chen BH Lee Chao PD Wan L Tsai FJ Puerariae radix isoflavones and their metabolites inhibit growth and induce apoptosis in breast cancer cells Biochem Biophys Res Commun 2009 378 683 688 10.1016/j.bbrc.2008.10.178 19013426

[B14] Yu Z Li W Induction of apoptosis by puerarin in colon cancer HT-29 cells Cancer Lett 2006 238 53 60 10.1016/j.canlet.2005.06.022 16055262

[B15] Caan BJ Natarajan L Parker B Gold EB Thomson C Newman V Rock CL Pu M Al-Delaimy W Pierce JP Soy food consumption and breast cancer prognosis Cancer Epidemiol Biomarkers Prev 2011 20 854 858 10.1158/1055-9965.EPI-10-1041 21357380 PMC7359724

[B16] Messina M Hilakivi-Clarke L Early intake appears to be the key to the proposed protective effects of soy intake against breast cancer Nutr Cancer 2009 61 792 798 10.1080/01635580903285015 20155618

